# Feasibility of Digital Image Correlation for Fatigue Cracks Detection under Dynamic Loading

**DOI:** 10.3390/s21196457

**Published:** 2021-09-27

**Authors:** Vladimir V. Bardakov, Artem Yu. Marchenkov, Anton Yu. Poroykov, Alexander S. Machikhin, Milana O. Sharikova, Natalya V. Meleshko

**Affiliations:** 1 Moscow Power Engineering Institute, 14 Krasnokazarmennaya, 111250 Moscow, Russia; bardakovvv@interunis-it.ru (V.V.B.); marchenkovay@mpei.ru (A.Y.M.); poroykovay@mpei.ru (A.Y.P.); sharikova.mo@ntcup.ru (M.O.S.); meleshkonv@mpei.ru (N.V.M.); 2 Scientific and Technological Center of Unique Instrumentation, Russian Academy of Sciences, 15 Butlerova, 117342 Moscow, Russia; 3 Railway Research Institute, 10, 3rd Mytischinskaya, 129626 Moscow, Russia

**Keywords:** image processing, digital image correlation, finite element method, fatigue cracks

## Abstract

We address non-contact detection of defects in the railway rails under their dynamic loading and propose to combine digital image correlation (DIC) and finite element modeling (FEM). We show that accurate model of defect-free rail operating at the same loading conditions as the inspected one provides a reliable reference for experimental data. In this study, we tested the rail samples with artificial and fatigue defects under cyclic loading, calculated displacement and stress distributions at different locations of the cracks via DIC and validated the obtained results by FEM. The proposed DIC-FEM approach demonstrates high sensitivity to fatigue cracks and can be effectively used for remote control of rails as well as for non-destructive testing of various other objects operating under dynamic loads.

## 1. Introduction

Rails are essential and the most loaded part of the railway transport system. They operate under conditions of intense cyclic loads that results in contact surface staining and fatigue damage, which may cause a train derailment [1]. Constant increasing train speed and traffic leads to the growing railway stresses. Modern typical rail flaws transform from manufacturing and welding defects to fatigue deformations and cracks [2,3,4]. Timely detection of fatigue damage and tracking its development kinetics provide trouble-free operation and reduce traffic accident rate. To ensure reliable detection of defects in rails at an early stage, non-destructive testing (NDT) methods are necessary [5,6].

Rail surface and subsurface deformation detection appears to be challenging for NDT inspection due to the wide range of flaw shapes and orientations [1,7]. Though conventional ultrasonic, eddy current and magnetic NDT techniques are quite effective, they do not ensure 100% defect detection due to human errors when interpreting the results, tricky shape of the rail and omission of unfavorably located crack-like defects [8,9,10,11]. Thus, the reliable and accurate flaw detection normally requires applying a few NDT techniques simultaneously [5,6,12,13,14]. A variety of methods which have been applied to analyze various factors effecting the reliability of defect detection in rails and absence of universal commercially available solutions confirm that NDT inspection still remains an important task.

With regard to the stress analysis of rails under train loads, optical techniques seem to be especially attractive as they provide non-contact, high-speed and accurate measurements [15,16,17]. Among these techniques, digital image correlation (DIC) is one of the most effective. It allows real-time micron-resolution two- and three-dimensional (3D) deformation mapping based cross-correlation image processing [18]. DIC has been already applied to the railroad tie inspection [19], rail displacements measurement [20] and other tasks related to rail studies.

In this study, we discuss DIC feasibility for solving one more NDT task related to railway rails—quantitative characterization of surface fatigue cracks. The main obstacle is the absence of reference data, i.e., stress fields of defect-free rails obtained at the same loading conditions as the inspected ones. To overcome this, we propose to model the ground truth data with respect to real geometrical and mechanical properties of the inspected rail as well as its loading mode. For this purpose, we made a series of thin rail samples with various locations of defect concentrators, grew fatigue cracks by its cycle loading, measured stress distribution using DIC and validated the experimental results by finite element modeling (FEM). Though the conditions of this laboratory study are quite far from the practice, obtained results clearly indicate the feasibility of the proposed DIC-FEM approach and necessity of its further development.

## 2. Experimental Protocol

### 2.1. Samples Preparation

**Figure 1:**
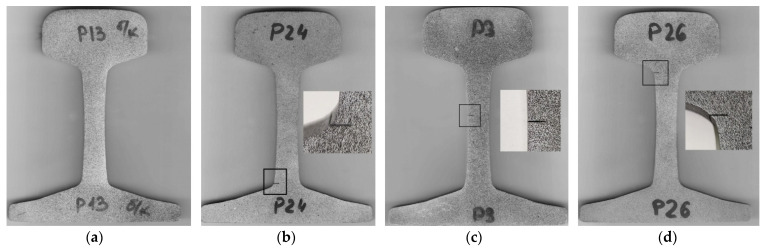
Samples of the rail sections without (**a**) and with incision in the foot (**b**), web (**c**) and head (**d**).

For our experiments, we have used the rail type R43 widely spread in railway industry. It is made of high-carbon steel E76F (analog to DIN steel grade 1.1625) and has the following chemical composition (wt., %): C—0.71–0.82, Si—0.25–0.60, Mn—0.75–1.15, S—≤0.025, P—≤0.025, V—0.03–0.15, Al—≤0.02. Mechanical properties of this steel were estimated by tension test: elastic limit *R*_e_ = 465 MPa, yield stress *R*_0.2_ = 540 MPa, ultimate tensile stress *R*_u_ = 1048 MPa, and total elongation 7.4%. From this rail, we cut 9 sections with 10 mm thickness and polished surfaces by a grinding machine. A total of 6 of these samples were incised in order to imitate the defect concentrator. By electroerosion machining, we made horizontal incisions with 4 mm depth and 0.25 mm width in the foot (2 samples), web (2 samples) and head (2 samples) of the rail (Figure 1).

**Figure 2:**
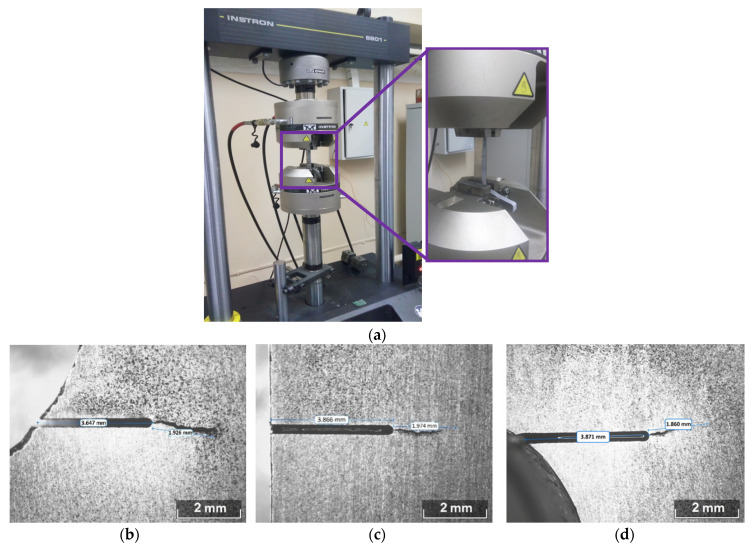
Sample installed in the testing machine (**a**) and images of the cracks grown in the foot (**b**), web (**c**) and head (**d**) of the sample.

Three of incised samples were subjected to cycle loading in order to grow fatigue cracks. For this, we installed each of these samples in the grips of the testing machine Instron 8801 and applied a smooth cyclic stretching loading with sinusoidal cycle of 10 Hz frequency and 250 MPa average stress across the section at maximal load (Figure 2a). We controlled the dimensions of the cracks via microscope interactively trying to provide its similar lengths of about 2 mm in the foot (Figure 2b), web (Figure 2c) and head (Figure 2d).

Thus, we have obtained 3 types of samples: defect-free (3 pcs), with incisions (3 pcs), and with incisions and cracks (3 pcs). Appropriate operation of DIC machine vision system requires a high-contrast surface texture. As a polished metal does not have it, we have created a random pattern on one of the samples’ surfaces by white and black spray paint.

### 2.2. Experimental Setup

**Figure 3:**
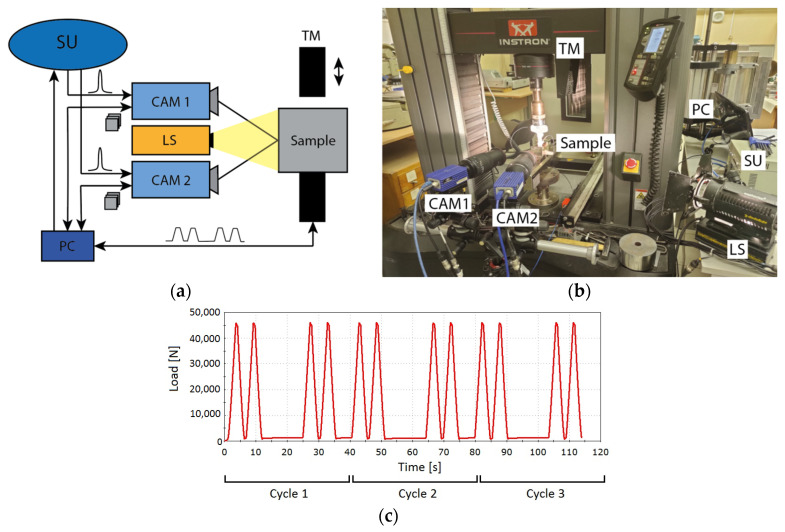
Scheme (**a**) and appearance (**b**) of the experimental setup, and loading cycles (**c**).

Experimental setup is based on testing machine Instron 5982 (TM) which is necessary to imitate a compressive cyclic loading caused by the movement of three train cars. With respect to the weight 36,000 kg, we applied 45 kN maximum force with a duration equal to 1 s (Figure 3c). Each sample was installed in TM, subjected to this loading, illuminated by 150 W white-light halogen light source LS (Dedolight DLH4-300) and observed by the machine vision system (LaVision StrainMaster) (Figure 3a,b). This system consists of two identical monochrome CCD cameras CAM1 and CAM2 (Basler piA2400-17gm) with 2456 × 2058 2/3” image sensors, synchronization unit SU (LaVision PTU) for simultaneous image acquisition, and a personal computer PC with DaVis 8.4 software for data acquisition, storage and processing. Both cameras are equipped with zoom lenses (Canon EF-S 12–200 mm f/3.5–5.6 IS). Each experiment included 2-min acquisition of 2300 × 1300 12-bit images of 30 × 20 mm^2^ sample’s area at 20 fps and joint processing of these images using DIC.

### 2.3. Data Processing

The images were processed using LaVision Davis StrainMaster software. To carry out 3D geometrical measurements and to reduce the effect of optical aberrations, the machine vision system must be calibrated in terms of cameras and their relative position. After the cameras CAM1 and CAM2 are positioned properly and provide crisp images of the inspected area, a calibration test-chart (black circles on a white background) is placed instead of the sample. The software automatically determines the circles on the target and calculates the calibration parameters [21].

After calibration, reference images of the unloaded sample have to be captured. When TM loads the sample, we analyze surface displacements by comparing the reference and subsequent images. For displacement detection, a least squares matching (LSM) algorithm [22] is applied with the following parameters: subset 31 × 31 pixels, Step 8 pixels, maximal expected deformation between two consecutive images 200 pixels. It is based on the optimization of the affine transformation parameters in terms of maximal coincidence of local image elements. The measured displacement distribution may be used to calculate to maps of material strain, and the Poisson ratio, etc. [18].

## 3. Finite Element Modeling

**Figure 4:**
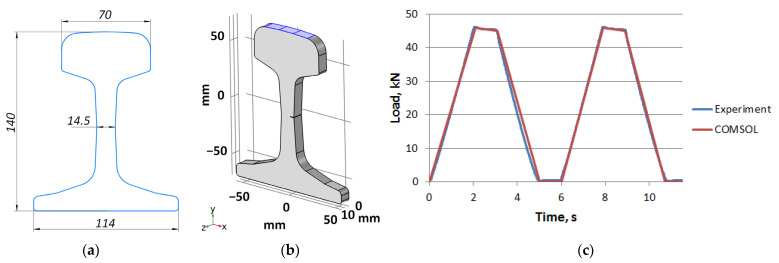
FEM modeling: front view drawing (**a**), 3D model of the sample (**b**) and loading curve (**c**).

To validate DIC measurements, we have simulated all experiments via FEM in COMSOL Multiphysics [23]. For simulating the load-induced displacement distribution for the entire loading cycle, we applied time-domain calculations using the Solid Mechanics module with the same repetition rate 20 Hz as DIC measurements. To ensure adequate simulation conditions, 3D shape of each sample and loading curve in every experiment were measured and imported to the simulation software. This data for one of the experiments is shown in Figure 4. Locations and dimensions of the incisions as well as rail steel properties (Young’s modulus 210 GPa, density 7800 kg/m3, Poisson’s ratio 0.3) were assigned in the FEM model in strict accordance with the real data. The rail was modeled as a linear elastic material.

The mesh for calculations consisted of 21,148 tetrahedra, 4270 triangles, 587 edge and 72 vertex elements. The load was applied to the top of the sample. The bottom boundary of the rail was fixed while other boundaries of the rail were free. To calculate the sample’s displacement vector, **u**, under the load, the equation of motion has to be solved in each mesh element [24]:

(1) $$\rho \frac{\partial^{2}u}{\partial t^{2}}=F_{V}-\nabla \cdot P^{T}$$

where ρ is density of the material, $\nabla \cdot P^{T}$ is gradient of the Piola–Kirchhoff stress tensor *P*, and $F_{v}$ is the volume force vector. After u*_i_* is found for each *i*-th (*i =* 1,2...*N*) element, its train *S_i_*, i.e., relative displacement u*_i_*/u_0_ of the Points P1*_i_* and P2*_i_* in the loaded (at the moment *t*) and unloaded (at the moment *t*_0_) conditions, may be found:

(2) $$S_{i}=\frac{\left|u_{i}\right|}{\left|u_{0}\right|}=\frac{P2_{i}\left(t\right)-P1_{i}\left(t\right)}{P2_{i}\left(t_{0}\right)-P1_{i}\left(t_{0}\right)}.$$

After **u***_i_* and *S_i_* are computed in each *i*-th mesh element, 3D displacement *u_FEM_(x,y,z)* and strain *S_FEM_(x,y,z)* fields may be built and compared with the distributions *u_DIC_(x,y,z)* and *S_DIC_(x,y,z)* calculated from DIC data.

## 4. Comparison of Experimental and Modeling Data

**Figure 5:**
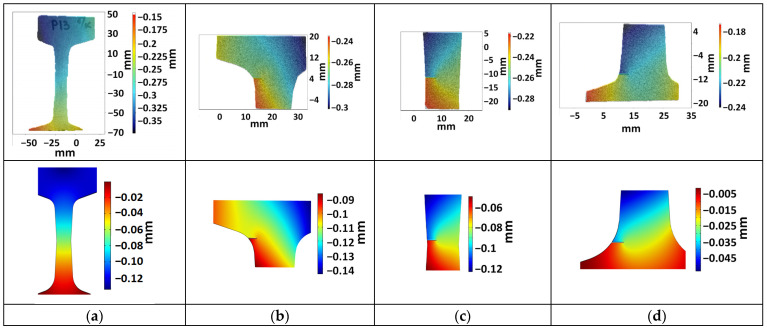
Displacement distributions obtained by DIC (upper row) and FEM (lower row) for the sample without incision (**a**) and with incision in the head (**b**), web (**c**) and foot (**d**) of the rail.

In all experiments, displacement and strain distributions were obtained both using DIC and via modeling. Figure 5 illustrates the displacement fields *u_DIC_(x,y,z)* and *u_FEM_(x,y,z)* for one sample without incisions and three samples with them at the moment *t* of the maximal load 45 kN. For all these crack-free samples, our studies demonstrate a good correspondence between experimental DIC data and FEM modeling.

Displacement values from FEM and DIC differ from each other in absolute values due to the fact that in the experiment, elastic deformations occur not only in the rail but also in the elements of the testing machine. In particular, the sample is located on the lower support, which is also elastically compressed during testing. As a result, the system including the rail undergoes additional parasitic downward displacements, which are about 0.15–0.17 mm and are not considered during FEM.

**Figure 6:**
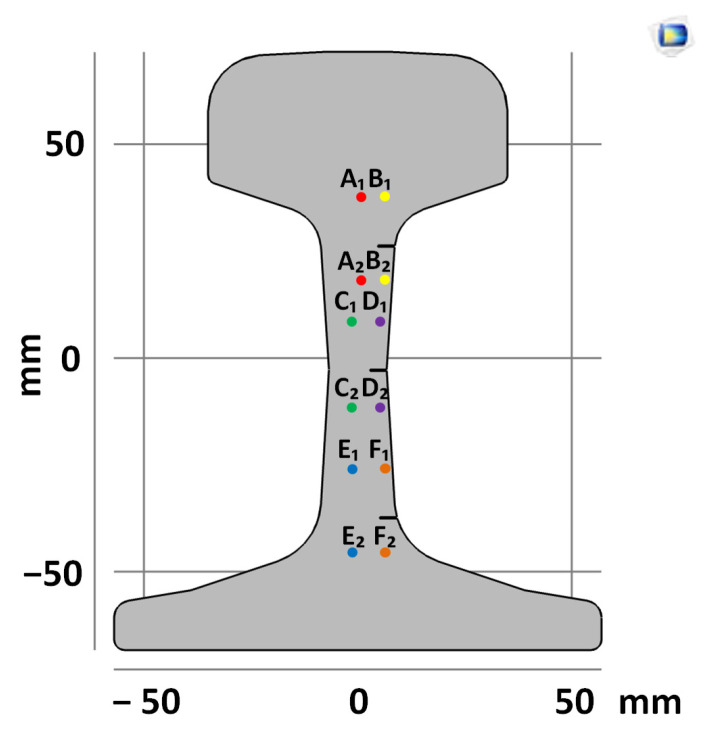
Model of the sample with points for comparative analysis of DIC and FEM.

To analyze the difference between DIC and FEM measurements in the absence and in the presence of the fatigue cracks, we have selected six pairs of points for strain measurements between them (Figure 6). In the head, web and foot of the rail, there are two pairs of points located in the incision area (B, D and F) and out of it (A, C and E).

Table 1 shows temporal dependencies of strain S in these points for the samples with and without the crack. For crack-free samples, DIC and FEM measurements are close (left column in Table 1). When the crack appears, difference between experimentally measured strain and the same FEM values becomes obvious (right column in Table 1). For Points A, C and E in the crack area this difference is smaller significantly than for Points B, D and F in the incision area. In all cases (Points A–F), the strain curves after unloading return to zero values, which confirms the elastic nature of the sample deformation.

For quantitative characterization, we have calculated the maximal $\Delta S_{max}$, average $m_{\Delta S}$ and standard deviation $\sigma_{\Delta S}$ values of the difference in strain $\Delta S=S_{DIC}-S_{FEM}$ between DIC and FEM data (Table 2):

(3) $$\begin{array}{l} \Delta S_{\max}=\max\left(\left|S_{\mathrm{DIC}}-S_{\mathrm{FEM}}\right|\right),\\ m_{\Delta S}=\frac{1}{N}\sum_{i=1}^{N}\left(\left|S_{\mathrm{DIC}i}-S_{\mathrm{FEM}i}\right|\right),\\ \sigma_{\Delta S}=\sqrt{\frac{1}{N-1}\sum_{i=1}^{N}{\left(\left|S_{\mathrm{DIC}i}-S_{\mathrm{FEM}i}\right|-m_{\Delta S}\right)}^{2}}. \end{array}$$

We may see that all these three parameters are sensitive to the presence of the crack. Crack causes the increase of $\Delta S_{max}$ by 1.8–3.9 times, $m_{\Delta S}$ by 1.3–6.8 times and $\sigma_{\Delta S}$ by 1.8–3.5 times. In fact, even in the area without incision (Points A, C and E), these values also increase by 1.8–2.3 times, $m_{\Delta S}$ by 1.4–3.3 times and $\sigma_{\Delta S}$ by 1.8–3.4 times, correspondingly. Such a high sensitivity of ΔS to the crack gives a reason to formulate quantitative criterions for the presence of a crack. In the simplest case, exceeding the threshold values of these or other parameters related to ΔS may indicate the presence of the defect not considered by FEM. In practice, for high defect detection probability, more complicated decision-making rules are necessary with respect to possible orientations, locations and dimensions of the cracks, wide range of loading conditions and other factors.

## 5. Conclusions

DIC has become a powerful tool for 3D displacement mapping widely used in mechanical engineering and NDT applications. Being full-field and non-contact, this method is effective for measuring contour, deformation, vibration and strain on various materials. To make this method applicable for defect detection, DIC-based displacement maps have to be compared with ground-truth “defect-free” images obtained for the same object in the same conditions. This is barely possible because of a priori unknown locations and dimensions of the defects as well as complicated shape which may vary from sample to sample. In the case of the railway rails, this concept becomes even more difficult to implement due to the need of identical dynamic loading for reference DIC measurement.

In this study, we have demonstrated that accurate finite element model built with respect to real geometrical and mechanical properties of the sample and real loading conditions provides quite reliable reference displacement distributions.

Comparing DIC data from the inspected rail under dynamic loading with this reference allows detection of significant deviations indicating the presence of defects, which are not included in the mathematical model. As soon as crack appears, we immediately see the difference between the experimental data and FEM simulation. Our experiments show that DIC-based strain values stably exceed FEM-based ones in the presence of fatigue cracks.

In this study, we have verified the proposed method on the thin sections of rail in laboratory conditions using a testing machine. This is the first step towards its practical application. Real conditions are quite different from the laboratory and require applying DIC to the side surface of a rail. To implement DIC-FEM approach in practice, much more factors have been analyzed and taken into account. That is why further research has to include the study of full-length rails and analyzing the metrological aspects of this technique. We believe that combined DIC-FEM approach may be quite effective as for remote control of rails so for NDT of other industrial objects operating under dynamic loading.

## Figures and Tables

**Table 1:** Temporal dependencies of strain in Points A–F (Figure 6) obtained by DIC (blue) and FEM (red).

Point	Sample	
	with Incision and without Crack	with Incision and with Crack
A	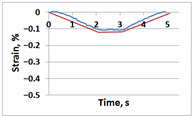	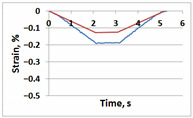
B	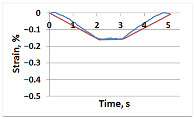	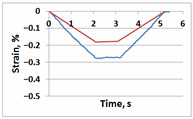
C	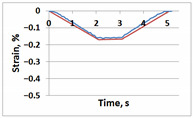	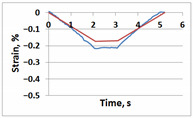
D	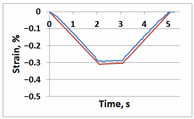	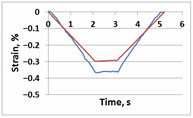
E	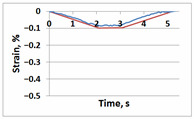	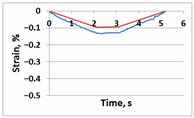
F	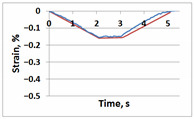	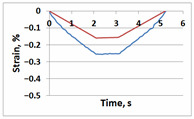

**Table 2:** Comparison of DIC and FEM measurements.

Sample		Points	Δ*S*_max_⋅10^−3^, %	*m*_Δ*S*_⋅10^−3^, %	*σ*_Δ*S*_⋅10^−3^, %
Incision	Crack				
In head	NO	A	30	20.0	6.1
		B	40	23.2	9.8
In web	NO	C	30	16.6	6.2
		D	36	24.7	6.7
In foot	NO	E	21	8.7	6.3
		F	27	10.8	8.9
In head	YES	A	70	32.0	21.1
		B	92	54.1	27.2
In web	YES	C	53	23.0	18.0
		D	73	31.0	23.7
In foot	YES	E	45.2	29.1	11.4
		F	104	73.0	26.1
